# Oral-Functioning Questionnaires in Patients with Head and Neck Cancer: A Scoping Review

**DOI:** 10.3390/jcm12123964

**Published:** 2023-06-10

**Authors:** Matthijs In ’t Veld, Derk H. J. Jager, Chayenne N. Chhangur, Kirsten A. Ziesemer, Frank K. J. Leusink, Engelbert A. J. M. Schulten

**Affiliations:** 1 Department of Oral and Maxillofacial Surgery/Oral Pathology, Amsterdam UMC and Academic Centre for Dentistry Amsterdam (ACTA), Vrije Universiteit Amsterdam, De Boelelaan 1117, 1081 HV Amsterdam, Noord-Holland, The Netherlands; d.jager@amsterdamumc.nl (D.H.J.J.); c.chhangur@amsterdamumc.nl (C.N.C.); f.leusink@amsterdamumc.nl (F.K.J.L.); eajm.schulten@amsterdamumc.nl (E.A.J.M.S.); 2 Amsterdam UMC, Vrije Universiteit Amsterdam, Medical Library, De Boelelaan 1117, 1081 HV Amsterdam, Noord-Holland, The Netherlands; k.a.ziesemer@amsterdamumc.nl

**Keywords:** head and neck cancer, oral functioning, quality of life, questionnaires, radiotherapy

## Abstract

**Background:** Oral-functioning impairment can negatively affect the quality of life (QoL) of head and neck cancer (HNC) patients after receiving radiotherapy (RT). Assessment of patient-reported oral functioning throughout treatment can improve patient care. This scoping review aims to propose a definition for oral functioning for HNC patients and to map out the available questionnaires measuring patient-reported oral functioning in RT-treated HNC patients. **Methods:** A literature search in relevant databases was performed. Each questionnaire was scored on the domains validity, reliability, and responsiveness. Furthermore, the items from the questionnaires were analyzed to define the common denominators for oral functioning in HNC patients. **Results:** Of the 6434 articles assessed, 16 met the inclusion criteria and employed 16 distinct instruments to evaluate QoL. No questionnaire covered all oral-health-related QoL items nor assessed all aspects of validity, reliability, and responsiveness. Chewing, speaking, and swallowing were the common denominators for oral functioning. **Conclusions:** Based on the included studies, we suggest using the VHNSS 2.0 questionnaire to assess oral functioning in HNC patients. Furthermore, we suggest to more clearly define oral functioning in HNC patients by focusing on masticatory function (chewing and grinding), mouth opening, swallowing, speaking, and salivation.

## 1. Introduction

Head and neck cancer (HNC) refers to a group of cancers occurring in the head and neck region, including the mouth, throat, nose, sinuses, and larynx. HNC can be caused by a variety of factors, including tobacco and alcohol use, exposure to certain chemicals, and certain types of human papillomavirus (HPV). HNC treatment consists of multiple pathways and requires a multidisciplinary approach. For patients with oropharyngeal, hypopharyngeal, or laryngeal squamous cell carcinoma, the diagnosis and staging is completed after examining the clinically and radiologically suspected sites during panendoscopy. The curative treatment of choice may include ablative surgery with or without (adjuvant) radiotherapy (RT) or chemoradiation [1].

Conventional RT causes several well-known complications in the head and neck region, including dysphagia, xerostomia, trismus, and mucositis [2]. Although most of these complications are not associated with an immediate risk of death, they may impact the patients’ quality of life (QoL) as they prolong states of pain and cause functional, aesthetic, nutritional, and psychological problems [3]. Intensity-modulated radiotherapy (IMRT) was introduced in 2007 and particularly aimed to reduce radiation doses to adjacent organs at risk, including the mucosa, salivary glands, and muscle tissue of the larynx and pharynx [4]. As a result, a decrease in adverse effects, such as dysphagia and xerostomia, has been observed from IMRT compared to conventional RT [5,6]. However, acute and long-term side effects after IMRT treatment remain prevalent, potentially affecting the QoL in HNC patients [7].

In recent years, the use of QoL questionnaires has gained popularity in the evaluation of cancer treatment, with increasing interest in oral functioning [3,8]. Resulting from disease as well as treatment, HNC patients may experience significant changes in oral functioning, including problems with speech, eating, swallowing, breathing, and changes in taste and smell [9]. A decrease in oral functioning can negatively affect the QoL in HNC patients. In order to optimize patient care and satisfaction throughout treatment, data regarding oral functioning is of eminent importance in RT-treated HNC patients. Such data can aid in identifying and prioritizing the most pressing oral health needs of the patient and in developing a plan for addressing these needs through appropriate treatment and care. It is therefore required that oral functioning is included in QoL questionnaires that target RT-treated HNC patients. A valid and reliable measuring instrument is crucial to record patients’ self-reported oral functioning.

In the literature, there is no clear consensus regarding the definition of oral functioning in HNC patients. Oral functioning is a very broad concept, which makes the term vague. Oral functioning or oral functions can be described, for example, as tongue pressure, tongue–lip motor function, and occlusal force [10]. However, oral functioning can also be divided into different spectrums, such as vital functions and social functions [11]. In addition, oral functioning can also be described in other domains, such as myo-functional activities, sensory functions, and social functions [12]. To properly document patient-reported oral functioning in HNC patients, a uniform and clear definition of the term is desirable.

The aim of this scoping review is to propose a definition for oral functioning and to map out the currently available questionnaires measuring patient-reported oral functioning in HNC patients treated with RT. Due to the lack of consensus regarding the definition of oral functioning, we suggest a new demarcated definition based on the items scored in the included questionnaires. In addition, we aim to identify which particular domains of oral functioning are addressed in the available questionnaires.

## 2. Material and Methods

A scoping review was conducted in accordance with the Cochrane Handbook for Reviews of Interventions and followed the guidelines provided in the Preferred Reporting Items for Systematic Reviews and Meta-Analyses (PRISMA) statement [13].

### 2.1. Search

In collaboration with an information specialist of the medical library (K.A.Z.), a comprehensive search was performed in the following bibliographic databases: PubMed, Embase, Clarivate Analytics/Web of Science Core Collection, and Wiley/Cochrane Library. Databases were searched for relevant literature from inception to 15 March 2021. The following search terms, including synonyms, closely related words, and keywords, were used as index terms or free-text words: “head and neck cancer”, “intensity modulated radiotherapy” and “quality of life questionnaire”. The searches contained no methodological search filter, date, or language restrictions. Duplicate articles were excluded using Endnote (X9.3.3, AED and Bramer methods, Clarivate Analytics, Philadelphia, PA, USA). The full search strategy for all databases can be found in Table S1.

### 2.2. Selection Criteria

Articles were included if they met the following criteria: (1) questionnaires for HNC patients (larynx, hypopharynx, oropharynx, nasopharynx, oral cavity, tongue, floor of mouth) who received radiotherapy or intensity-modulated radiotherapy; (2) published before 1 April 2021; (3) published in English, and (4) questionnaires assessing multiple domains of oral functioning. Articles were excluded for the following reasons: (1) not original quantitative or qualitative research articles (e.g., case reports, editorials, letters to editor, oral papers and posters, conference abstracts); (2) patients with other malignancies than HNC; (3) animal or cadaveric studies; and (4) questionnaires assessing only a single domain of oral functioning (e.g., dysphagia-specific or speech-specific questionnaires).

### 2.3. Data Screening and Abstraction

Two reviewers (C.N.C. and M.V.) independently screened all potentially relevant titles and abstracts for eligibility. Preliminary screening was conducted in Rayyan software (Qatar Computing Research Institute, Doha, Qatar). Disagreements were resolved by discussion. If no consensus was reached, a third researcher (F.L.) resolved any disagreements. Endnote X9 (Thomson Reuters, New York, NY, USA) was used to organize references.

### 2.4. Definitions of Validity and Reliability

Validity is the extent to which an instrument measures what it is supposed to measure. It can be divided into four subgroups: criterion validity, construct validity, content validity, and clinical validity [14,15,16].

Criterion validity compares the results of a test with an external criterion, such as a gold standard, to determine the correlation between the outcome of the test and the gold standard. It is divided into concurrent validity, which compares the results of different questionnaires measuring the same concept. Predictive validity determines whether a test can accurately predict the results of another test. In this scoping review, predictive validity is disregarded/kept aside.

Content validity assesses whether the test items are relevant to the concept being measured. This can be determined by a layman or an analyst through face validity, which examines whether an instrument appears to measure something relevant. It can also be determined by a group of experts through expert panel validity. There is little distinction made between face validity and expert panel validity in practice.

Construct validity assesses whether a test measures what it is supposed to measure by comparing its results to those of another test that measures the same concept. This includes assessing convergent and discriminant validity, which measure the strength of the correlation between the two tests and whether they are measuring different concepts, respectively. Known groups validity is also used to assess construct validity by comparing test scores between groups that are expected to differ in the feature being measured. For example, this can be applied to head and neck cancer QoL questionnaires by comparing scores between different radiation techniques, stages, or ages.

Clinical validity is about the accuracy of a test to indicate a clinical condition. This can be described by the following conceptions: sensitivity, specificity, and positive- and negative-likelihood ratio.

Reliability refers to the consistency and stability of test results over time. It can be measured in different ways, such as test–retest reliability and internal consistency [17,18,19].

Test–retest reliability measures the consistency of results when the test is repeated under the same conditions. Pearson correlation coefficient, Cohen’s Kappa, and interclass correlation coefficient are commonly used measures. A score of less than 0.5 indicates poor reliability, 0.5–0.75 is moderate, 0.75–0.90 is good, and 0.90 is excellent.

Internal consistency measures the similarity of results among different parts of the test. This is measured by Cronbach’s alpha, with a score of greater than 0.70 being acceptable. Responsiveness is the ability of the instrument to detect clinically significant changes over time.

## 3. Results

**Figure 1:**
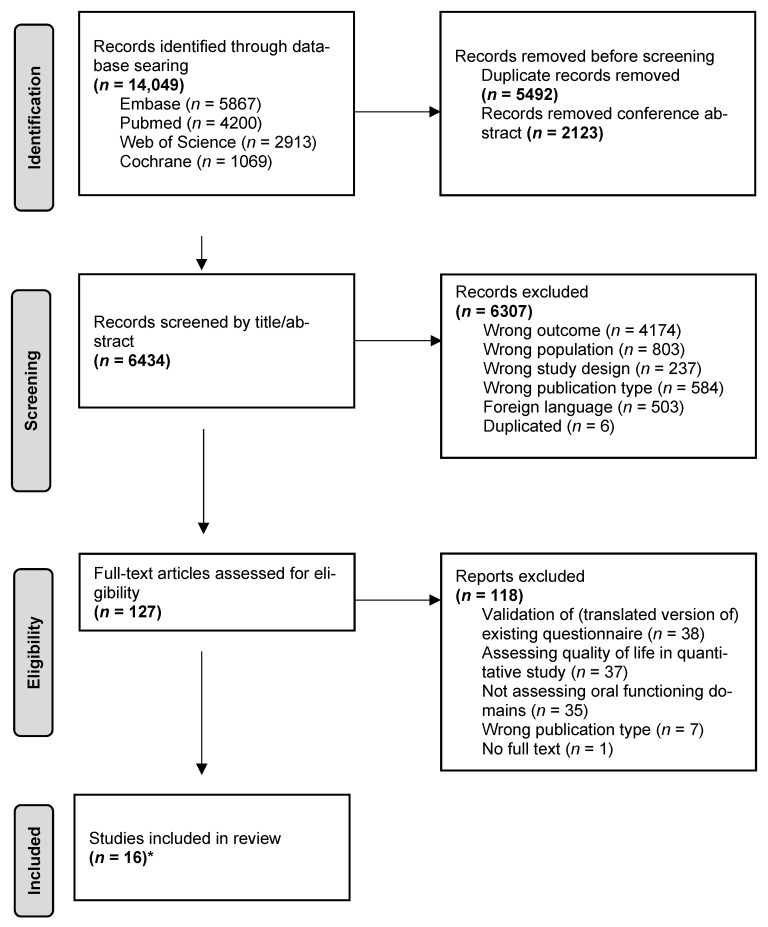
PRISMA flowchart of the article selection process. * Seven articles were included by a hand search in the reference lists.

The initial literature search yielded a total of 14.049 references: 4200 in PubMed, 5867 in Embase, 2913 in Clarivate Analytics/Web of Science Core Collection, and 1069 in Wiley/Cochrane Library. After removing duplicates of references that were selected from more than one database, 6434 references remained. On the basis of title and abstract, 6307 articles were excluded, and the full text of 127 articles was obtained for further consideration. Seven more references were found by a hand search. Sixteen articles met the inclusion criteria regarding questionnaires evaluating oral functioning in head and neck cancer patients. In Figure 1 the flowchart regarding the literature search and study selection process is presented.

### 3.1. Quality of Life Questionnaires with Oral Functioning Domains

Table 1 presents a summary of the OHRQoL items that have been covered in the questionnaires.

The FACT-H&N questionnaire is designed to assess four general domains of quality of life [20]. The scores for each domain can have different interpretations; for some, a higher score indicates a better quality of life, while for others, a higher score indicates a poorer quality of life.

The UW-QoL (version 4) questionnaire consists of 12 specified questions [21]. The questionnaire offers four, five, or six answer options, each corresponding to a range from 0 to 100. Lower numbers represent a lower QoL. Additionally, the questionnaire includes an additional question that allows the patient to indicate the most important domain.

The UW-QoL RTOG is a modified version of the UW-QoL questionnaire that addresses specific problems faced by patients undergoing radiotherapy [22]. Additional items have been included in this version of the questionnaire to assess pain, saliva production, and shoulder disability. The individual scores for each item are added together and then averaged to obtain the final score. A low final score indicates a high health-related quality of life, whereas a high final score suggests a decreased health-related quality of life.

The EORTC QLQ-C30 questionnaire contains multiple domains [23]. To standardize the scores, all scores are linearly transformed so that they range from 0 to 100. In the functional and overall quality of life domains, high scores indicate a good quality of life. In the symptom scale, a high score indicates a higher symptom burden and therefore a lower quality of life.

The EORTC QLQ-H&N35 has been specifically designed to target patients with HNC [24]. It contains 35 questions, divided into 18 different domains. A high score on the questionnaire indicates a lower quality of life.

The treatment protocols for HNC have undergone changes in the past decades. As a result, the EORTC QLQ-H&N35 questionnaire was revised and updated to the EORTC QLQ-HN43 [25]. The questionnaire assesses the same domains as the EORTC QLQ-H&N35, with the exception of malaise, nutritional supplements, painkillers, and tube feeding. Additionally, the EORTC QLQ-H&N43 also evaluates emotional well-being as well as hand/feet, shoulder, and skin issues.

The EORTC QLQ-OH15 questionnaire is a tool specifically designed to assess OHRQoL in patients with cancer [26]. The scoring procedure is similar to that of the EORTC QLQ-C30. A higher score on the information scale and the OHRQol scale indicates higher levels of functioning and information, whereas a higher score on the symptom scale indicates a greater degree of symptoms or problems. The QLQ-C30 and QLQ-OH15 questionnaires have been combined and named QLQ-OH45.

The EORTC QLQ-OH17 questionnaire was developed as an additional module to the EORTC QLQ-C30 [27]. It is also a tool specifically designed to assess OHRQoL in cancer patients. The scoring procedure for the QLQ-OH17 is similar to that of the EORTC QLQ-C30 and the EORTC QLQ-OH15, as described above.

The VHNSS 1.0 questionnaire is designed to assess various functional domains related to OHRQoL in patients with HNC [28]. The items are scored on a scale from 0 to 10, where 0 represents “none” and 10 represents “severe”. A lower score indicates fewer symptoms and therefore a higher quality of life.

Although The VHNSS 1.0 questionnaire includes oral health outcomes, the tool does not cover all potential adverse effects of head and neck cancer therapy [29]. To address this, the questionnaire was expanded to include an oral health sub-scale that assesses a wider range of oral health outcomes as well as the functional implications of oral side effects. Similar to the VHNSS 1.0, the items are scored on a scale from 0 (none) to 10 (severe).

The EQ-5D-3L consists of two parts: the EQ-5D descriptive system and the EQ-5D visual analogue scale (EQVAS) [30]. A formula is applied that assigns values to each problem level in each domain, with a range from −0.111 (worse than death, severe problems in all five domains) to 0 (death) to 1 (completely healthy, no problems in all five domains).

The QOL-NPC (version 2) contains 33 items across various domains [31]. The questionnaire includes items related to oral health as well as non-oral health related items. The response of 1 represents “not at all” (excellent), 2 represents “a little bit” (very good), 3 represents “moderate” (good), 4 represents “quite a bit” (fair), and 5 represents “extreme” (poor).

The QOL-RTI/H&N consists of two components: the QOL-RTI and the H&N module [32]. The H&N module consists of 14 questions that specifically evaluate oral-health-related domains. The answers for all items in each domain are added together and then averaged to produce a single score. A higher score indicates a better quality of life.

The LORQ is a questionnaire used to assess OHRQoL in individuals with dentures or other oral rehabilitation needs [33]. Higher scores indicate a greater degree of difficulty or dissatisfaction.

The OHIP-14 is a tool to evaluate the impact of oral health on patients’ QoL and assesses the impact of oral health on seven different dimensions [34]. The total OHIP-14 score is calculated by summing up the scores for all items, with a higher score indicating a greater impact of oral health on patients’ QoL.

The FIGS is a questionnaire used to evaluate the functional status of a patients’ intraoral structures and its impairment [35]. The scale includes the following intra-oral structures: the teeth, lips, tongue, and jaw. The total FIGS score is calculated by summing up the scores for all items. A higher score indicates a greater impairment of the patients’ oral functional status. Table 2 and Table 3 provide the structure of the questionnaires and a summary of the general and non-oral-health-related items they contain.

### 3.2. Validity and Reliability of the Questionnaires

Validity and reliability are examined for all questionnaires. As described before, several components of validity and reliability can be assessed. None of the studies examined all components. The validity and reliability of the QoL-NPC questionnaire were tested on seven items. Similarly, the reliability and validity of the FACT-H&N, UW-QoL, EORTC QLQ-H&N35, EORTC QLQ-HN43, VHNSS 2.0, QoL-RTI/H&N, and LORQ were assessed on four items. In contrast, the EORTC QLQ-C30 and EQ5D-3L only assess their reliability and validity on one item. Table 4 summarizes the statistical methods used to assess validity, reliability, and responsiveness, and it indicates whether the questionnaire is validated for HNC patients and if any translated versions are available.

### 3.3. Items Assessing Oral Functioning

Based on the included questionnaires and the corresponding scored items, various domains can be identified. Vital functions of oral functioning, such as biting, chewing, and swallowing, are scored in most questionnaires. This also applies to sensory oral functioning, such as taste, pain, and xerostomia, which are scored in most questionnaires. The EORTC QLQ-C30, EQ-5D-3L, and FIGS address fewer than three of the 16 OHRQoL items in Table 1. The UW-QoL, UW-QoL RTOG, VHNSS 1.0, VHNSS 2.0, and QOL-RTI/H&N are questionnaires that assess chewing, swallowing, taste, and voice (speech). With the exception of LORQ and FIGS, all questionnaires include pain. The VHNSS 1.0, VHNSS 2.0, and QOL-RTI/H&N are questionnaires that contain more than 10 items for assessing oral functioning.

## 4. Discussion

The aim of this scoping review was to systematically examine the existing questionnaires that assess QoL in HNC patients, with a specific focus on evaluating their ability to adequately capture oral functioning. Additionally, because there is no consensus in the literature regarding the definition, we would like to propose a clear definition of oral functioning in patients with HNC in order to achieve clarity and uniformity.

HNC treatments, such as RT and surgery, can result in functional impairments in the oral region. RT may cause hyposalivation, xerostomia, dysphagia, and changes in taste perception [40,41]. IMRT was found to have similar acute side effects compared to conventional RT. However, patients receiving IMRT had significantly fewer late side effects, including xerostomia and dysphagia, than those receiving the conventional technique [6]. In a recent meta-analysis, it was found that patients treated with IMRT had less severe xerostomia and hyposalivation than patients treated with conventional radiotherapy, suggesting that IMRT may lead to an improved quality of life [9]. Surgery may lead to the loss of structures necessary for speech and swallowing, such as the larynx or part of the tongue, resulting in decreased oral functioning. The extent and nature of these functional impairments can vary depending on the location, stage, and treatment modality of the head and neck cancer.

Despite the evident impact of cancer treatments on oral functioning, there is currently no consensus in the literature regarding a standardized definition of oral functioning in HNC patients [7,42]. Different authors have proposed various ways to define oral functioning. For instance, Yap et al. categorized oral functioning into different domains, including vital functions (such as biting, chewing, digesting/tasting, swallowing, speaking, and breathing), social functions, and anti-social functions [11]. Minakuchi et al. described oral function examination in seven clinical conditions, such as oral hygiene, oral dryness, tongue pressure, tongue-lip motor function, occlusal force, masticatory function, and swallowing function [10]. Eriksen et al. divided oral functioning into several domains, including myo-functional activities (such as eating, chewing, biting, drinking, smoking, swallowing, sucking, licking, drooling, and breathing), sensory functions (such as taste, mechanical sensation, temperature, and pain), social functions (such as speaking, singing, playing wind instruments, laughing, and mimicry), sexuality (including kissing and oral sex), and individual identity (such as aesthetics, social status, cosmetics, and body art, including oral piercing and jewelry) [12]. In a recent study, different aspects of oral function were described as oral competence [43]. Furthermore, a multitude of studies have used different assessment tools or measures to evaluate oral functioning, further complicating efforts to establish a consistent and precise definition. Moreover, the absence of a consensus not only poses a challenge for researchers attempting to compare outcomes across studies, but it also holds significant importance for clinical practice. Furthermore, the existing HNC questionnaires do not comprehensively capture the different aspects of oral function. Traditional head and neck questionnaires lack specificity and sensitivity in evaluating the effectiveness of rehabilitation and oral function as they only include a limited number of items related to topics, such as chewing [33]. Based on the included questionnaires we would like to suggest a demarcated definition of oral functioning in HNC patients mainly focusing on the physical aspects, including chewing, hyposalivation, speaking, swallowing, and taste.

One of the limitations of this scoping review is that we excluded specific oral health questionnaires, such as those for xerostomia and dysphagia, from our literature search. Although there are several questionnaires available to assess specific aspects of OHRQoL, such as the MD Anderson Dysphagia Inventory (MDADI) or radiotherapy-induced xerostomia questionnaire, which evaluates swallowing-related QoL, oral functioning encompasses multiple aspects, including masticatory functions, aesthetics/satisfaction/psycho-sociability, occlusal support/dental arch stability, xerostomia, and other oral functions, such as taste and pronunciation during speech [44,45]. Since these specific questionnaires only evaluate one aspect of oral functioning, they cannot comprehensively measure oral functioning. A second limitation of this study is that the literature search ended on 15 March 2021, which implies that more recent studies on oral functioning questionnaires in HNC patients might have been missed. Another limitation of this scoping review is that due to the significant heterogeneity among the articles included, a meta-analysis could not be conducted. Additionally, the quality of evidence was not assessed, which is not always necessary for a scoping review [46]. However, it is important to consider this lack of quality assessment when interpreting the results. Other limitations of this scoping review should also be acknowledged. While multiple databases were searched, including PubMed, Embase, Clarivate Analytics, Web of Science Core Collection, and Cochrane Library, searching additional databases, such as ScienceDirect or Google Scholar, could potentially yield more relevant studies.

In the future, to optimize patient care, there is a need to develop new oral-functioning questionnaires which are specific for HNC patients. These questionnaires should aim to incorporate all relevant and specific domains of oral functioning. Furthermore, the questionnaire should be easy to read, to understand, and to complete with clear instructions. Additionally, the length of the questionnaire should be limited. For example, a 35-item questionnaire could be perceived as lengthy by patients, which could lead to frustration by patients during completion of the questionnaires and could therefore influence the validity, reliability, and responsiveness [47,48]. Therefore, it is important to avoid numerous unnecessary lengthy questionnaires. The optimal time a patient is willing to dedicate to completing a questionnaire is 10 min [29,33]. In addition, a newly developed questionnaire should not include general characteristics which are not related to oral functioning, for example, weight gain or loss, malaise, insomnia, and use of nutritional supplements. This can improve the accuracy of the assessment and prevent confusion for the patient. The authors believe that combining patient-reported-outcome measures (PROMs) with specialists-reported-outcome measures (SROMs) could lead to a more comprehensive questionnaire evaluating oral functioning in HNC patients. Whereas PROMs focus on patients’ subjective experiences (e.g., chewing, grinding, speaking, and swallowing), SROMs focus on objective clinical measures (e.g., hyposalivation, mouth opening, and range of motion). By combining these approaches, the questionnaire captions both patients’ experiences and specialists’ clinical evaluation, providing a more complete assessment of oral functioning and allowing communication between patients and healthcare providers as it ensures both the patients’ concerns and clinical findings.

## 5. Conclusions

The authors suggest developing a new oral functioning questionnaire combining SROMs with PROMs focusing on physical aspects and excluding items not related to oral functioning. Based on the included studies, we suggest using the VHNSS 2.0 questionnaire in assessing oral functioning in HNC patients.

Furthermore, we suggest more clearly defining oral functioning in HNC patients by focusing on physical aspects, such as masticatory function (chewing and grinding), mouth opening, swallowing, speaking, and salivation.

## Figures and Tables

**Table 1:** Overview of OHRQoL items of the questionnaires.

Instruments	FACT-H&N [20]	UW-QoL		EORTC QLQ					VHNSS		EQ-5D-3L [30]	QOL-NPC [31]	QOL-RTI/H&N [32]	LORQ [33]	OHIP-14 [34]	FIGS [35]
		UW-QoL [21]	UW-QoL RTOG [22]	EORTC QLQ-C30 [23]	EORTC QLQ-H&N35 [24]	EORTC QLQ-HN43 [25]	EORTC QLQ-OH15 [26]	EORTC QLQ-OH17 [27]	VHNSS 1.0 [28]	VHNSS 2.0 [29]						
Oral Health Related Items																
Appearance	X	X	X		X	X						X	X	X	X	
Chewing	X	X	X				X	X	X	X			X	X	X	X
Choke/gag									X	X						
Dental health/problems					X	X	X	X	X	X		X				
Dentures							X	X	X	X				X	X	
Dry mouth (xerostomia)	X				X	X	X	X	X	X		X		X		
Lack of appetite				X	X	X		X	X	X		X	X			
Opening mouth (trismus)					X	X				X		X		X		
Oral information							X	X						X		
Pain	X	X	X	X	X	X	X	X	X	X	X	X	X		X	
Radioactive rhinitis												X				
Saliva/mucus	X	X	X		X	X	X	X	X	X			X	X		
Sores							X		X	X				X		
Swallowing	X	X	X		X	X			X	X		X	X	X		X
Taste		X	X		X	X	X	X	X	X			X		X	
Voice (speech)	X	X	X		X	X			X	X		X	X	X	X	X

FACT-H&N, the Functional Assessment of Cancer Therapy-Head and Neck; UW-QOL, the University of Washington Quality of Life Questionnaire; UW-QoL RTOG Modification, the University of Washington Quality of Life Questionnaire-RTOG Modification; EORTC QLQ-C30, the European Organization for Research and Treatment for Cancer Quality of Life Questionnaire Core 30; EORTC QLQ-H&N35, the European Organization for Research and Treatment for Cancer Quality of Life Questionnaire Head and Neck 35; EORTC QLQ-H&N43, the European Organization for Research and Treatment for Cancer Quality of Life Questionnaire Head and Neck 43; EORTC QLQ-OH15, the European Organization for Research and Treatment for Cancer Quality of Life Questionnaire Oral Health 15; EORTC QLQ-OH17, the European Organization for Research and Treatment for Cancer Quality of Life Questionnaire Oral Health 17; VHNSS 1.0 and 2.0, the Vanderbilt Head and Neck Symptom Survey version 1.0 and version 2.0; EQ-5D-3L, the 3-level version of the EuroQol 5-dimension scale; QOL-NPC, the Quality of Life scale for nasopharyngeal carcinoma; QOL-RTI/H&N, Quality of Life Radiation Therapy Instrument with Head and Neck Companion Module, LORQ, Liverpool Oral Rehabilitation Questionnaire; OHIP-14, Oral Health Impact Profile-14; FIGS, Functional intraoral Glasgow scale.

**Table 2:** Overview of the general and not OHRQoL-related items of the questionnaires.

Instruments	FACT-H&N [20]	UW-QoL		EORTC QLQ					VHNSS		EQ-5D-3L [30]	QOL-NPC [31]	QOL-RTI/H&N [32]	LORQ [33]	OHIP-14 [34]	FIGS [35]
		UW-QoL [21]	UW-QoL RTOG [22]	EORTC QLQ-C30 [23]	EORTC QLQ-H&N35 [24]	EORTC QLQ-HN43 [25]	EORTC QLQ-OH15 [26]	EORTC QLQ-OH17 [27]	VHNSS 1.0 [28]	VHNSS 2.0 [29]						
General Items																
Cognitive performance				X												
Emotional well-being	X	X		X		X		X			X	X	X	X	X	
Functional well-being	X	X	X	X							X	X	X	X	X	
Physical well-being	X	X	X	X	X	X					X	X	X	X	X	
Social/family well-being	X			X	X	X						X	X	X	X	
**Not oral health related items**																
Alcohol consumption	X															
Breathing	X															
Constipation				X												
Cough					X	X				X		X	X			
Diarrhea				X												
Dyspnea				X												
Fatigue				X								X	X			
Hand/feet						X										
Headache												X				
Hearing									X	X		X				
Insomnia				X						X		X				
Loss/gain of weight					X	X			X	X		X				
Malaise	X				X											
Nasal bleeding												X				
Nausea	X			X									X			
Neck																
Nutritional supplements					X				X	X						
Painkillers		X	X		X				X	X						
Shoulder		X				X				X						
Skin						X						X				
Smell					X	X				X						
Smoking	X															
Tube feeding					X				X	X						
Vomiting				X												

FACT-H&N, the Functional Assessment of Cancer Therapy-Head and Neck; UW-QOL, the University of Washington Quality of Life Questionnaire; UW-QoL RTOG Modification, the University of Washington Quality of Life Questionnaire-RTOG Modification; EORTC QLQ-C30, the European Organization for Research and Treatment for Cancer Quality of Life Questionnaire Core 30; EORTC QLQ-H&N35, the European Organization for Research and Treatment for Cancer Quality of Life Questionnaire Head and Neck 35; EORTC QLQ-H&N43, the European Organization for Research and Treatment for Cancer Quality of Life Questionnaire Head and Neck 43; EORTC QLQ-OH15, the European Organization for Research and Treatment for Cancer Quality of Life Questionnaire Oral Health 15; EORTC QLQ-OH17, the European Organization for Research and Treatment for Cancer Quality of Life Questionnaire Oral Health 17; VHNSS 1.0 and 2.0, the Vanderbilt Head and Neck Symptom Survey version 1.0 and version 2.0; EQ-5D-3L, the 3-level version of the EuroQol 5-dimension scale; QOL-NPC, the Quality of Life scale for nasopharyngeal carcinoma; QOL-RTI/H&N, Quality of Life Radiation Therapy Instrument with Head and Neck Companion Module, LORQ, Liverpool Oral Rehabilitation Questionnaire; OHIP-14, Oral Health Impact Profile-14; FIGS, Functional intraoral Glasgow scale.

**Table 3:** Overview of the format of the questionnaires.

Instruments	FACT-H&N [20]	UW-QoL		EORTC QLQ					VHNSS		EQ-5D-3L [30]	QOL-NPC [31]	QOL-RTI/H&N [32]	LORQ [33]	OHIP-14 [34]	FIGS [35]
		UW-QoL [21]	UW-QoL RTOG [22]	EORTC QLQ-C30 [23]	EORTC QLQ-H&N35 [24]	EORTC QLQ-HN43 [25]	EORTC QLQ-OH15 [26]	EORTC QLQ-OH17 [27]	VHNSS 1.0 [28]	VHNSS 2.0 [29]						
Format																
Number of items	38	12	15	30	35	43	15	17	33/28	50	5	26	38	39	14	3
Answer type	4-point Likert scale	4-,5- or 6-point Likert scale	5-point Likert scale	4-point Likert scale + 7-point Likert scale	4-point Likert scale	4-point Likert scale	4-point Likert scale	4-point Likert scale + dichotomous response (yes/no)	0–10 Likert scale	0–10 Likert scale	3 levels: no problems, some problems, and extreme problems	4-point Likert scale + dichotomous response (yes/no)	0–10 Likert scale	4-point Likert scale	5-point Likert scale	5-point Likert scale

FACT-H&N, the Functional Assessment of Cancer Therapy-Head and Neck; UW-QOL, the University of Washington Quality of Life Questionnaire; UW-QoL RTOG Modification, the University of Washington Quality of Life Questionnaire-RTOG Modification; EORTC QLQ-C30, the European Organization for Research and Treatment for Cancer Quality of Life Questionnaire Core 30; EORTC QLQ-H&N35, the European Organization for Research and Treatment for Cancer Quality of Life Questionnaire Head and Neck 35; EORTC QLQ-H&N43, the European Organization for Research and Treatment for Cancer Quality of Life Questionnaire Head and Neck 43; EORTC QLQ-OH15, the European Organization for Research and Treatment for Cancer Quality of Life Questionnaire Oral Health 15; EORTC QLQ-OH17, the European Organization for Research and Treatment for Cancer Quality of Life Questionnaire Oral Health 17; VHNSS 1.0 and 2.0, the Vanderbilt Head and Neck Symptom Survey version 1.0 and version 2.0; EQ-5D-3L, the 3-level version of the EuroQol 5-dimension scale; QOL-NPC, the Quality of Life scale for nasopharyngeal carcinoma; QOL-RTI/H&N, Quality of Life Radiation Therapy Instrument with Head and Neck Companion Module; LORQ, Liverpool Oral Rehabilitation Questionnaire; OHIP-14, Oral Health Impact Profile-14; FIGS, Functional intraoral Glasgow scale.

**Table 4:** The statistical methods used to assess validity, reliability, and responsiveness per included questionnaire.

		Validity				Reliability			Responsiveness	Validated for HNC Patients	Translated Versions
		Criterion Validity	Construct Validity	Content Validity	Clinical Validity						
		Concurrent				Test–Retest Reliability	Internal Consistency	Split-Half Reliability			
**FACT-H&N** [20,36]	FACT-H&N		X	X			X		X	X	X
**UW-QoL**	UW-QoL [21,36]	X	X				X		X	X	X
	UW-QoL RTOG Modification [22]	X				X	X			X	
	EORTC QLQ-C30 [23,36]		X							X	X
**EORTC QLQ**	EORTC QLQ-H&N35 [24,36]	X	X				X		X	X	X
	EORTC QLQ-HN43 [25]		X			X	X		X	X	X
	EORTC QLQ-OH15 [26]	X	X			X	X		X	X	X
	EORTC QLQ-OH17 [27]	X	X			N.R.	X			X	X
**VHNSS**	VHNSS 1.0 [28]		X	X			X			X	
	VHNSS 2.0 [29,37]	X	X	X			X			X	X
**EQ5D-3L** [30,38]			X						X	X	X
**QoL-NPC** [31]		X	X	X		X	X	X	X	X	X
**QOL-RTI/H&N** [32]		X	X			X	X		N.R.	X	X
**LORQ** [33,39]			X			X	X		X	NR	X
**OHIP-14** [34]			X				X		X	X	X
**FIGS** [35]		N.R.	N.R.	N.R.	N.R.	N.R.	N.R.	N.R.	N.R.		

FACT-H&N, the Functional Assessment of Cancer Therapy-Head and Neck; UW-QOL, the University of Washington Quality of Life Questionnaire; UW-QoL RTOG Modification, the University of Washington Quality of Life Questionnaire-RTOG Modification; EORTC QLQ-C30, the European Organization for Research and Treatment for Cancer Quality of Life Questionnaire Core 30; EORTC QLQ-H&N35, the European Organization for Research and Treatment for Cancer Quality of Life Questionnaire Head and Neck 35; EORTC QLQ-H&N43, the European Organization for Research and Treatment for Cancer Quality of Life Questionnaire Head and Neck 43; EORTC QLQ-OH15, the European Organization for Research and Treatment for Cancer Quality of Life Questionnaire Oral Health 15; EORTC QLQ-OH17, the European Organization for Research and Treatment for Cancer Quality of Life Questionnaire Oral Health 17; VHNSS 1.0 and 2.0, the Vanderbilt Head and Neck Symptom Survey version 1.0 and version 2.0; EQ-5D-3L, the 3-level version of the EuroQol 5-dimension scale; N.R., not reported; QOL-NPC, the Quality of Life scale for nasopharyngeal carcinoma; QOL-RTI/H&N, Quality of Life Radiation Therapy Instrument with Head and Neck Companion Module; LORQ, Liverpool Oral Rehabilitation Questionnaire; OHIP-14, Oral Health Impact Profile-14; FIGS, Functional intraoral Glasgow scale.

## Data Availability

Not applicable.

## Supplementary Materials

The following supporting information can be downloaded at: https://www.mdpi.com/article/10.3390/jcm12123964/s1, Table S1: Search strategy for relevant databases.
