# Supplementary material for: Oral-Functioning Questionnaires in Patients with Head and Neck Cancer: A Scoping Review
Source: J Clin Med. 2023 Jun 10;12(12):3964. doi: 10.3390/jcm12123964 (PMC10299551; doi:10.3390/jcm12123964)
Supplement: Supplementary file 1 [file jcm-12-03964-s001.zip › jcm-2429917-supplementary.pdf]

Table S1. Search strategy for relevant databases

PubMed 4200 (15 March 2021)

Search	PubMed Query – March 15, 2021	Results
4	#1 AND #2 AND #3	4,200
3	(("Quality of Life"[Mesh] OR "Activities of Daily Living"[Mesh] OR "Health Status"[Mesh]) AND "Surveys and Questionnaires"[Mesh]) OR (("quality of life"[tiab] OR life qualit*[tiab] OR living qualit*[tiab] OR "quality of living"[tiab]) AND ("assess*"[tiab] OR "question*"[tiab] OR "survey"[tiab] OR "instrument"[tiab] OR "index"[tiab])) OR "QLQ-HN35"[tiab] OR "QLQ-C30"[tiab] OR "H&N35"[tiab] OR "UWQOLQ"[tiab] OR "Neck Survey"[tiab] OR "FACT-H&N"[tiab] OR "oral function*"[tiab] OR "masticatory function*" OR "swallow*"[tiab] OR "chew*"[tiab]	299,735
2	"Radiotherapy"[Mesh] OR "radiotherapy"[Subheading] OR "Radiosurgery"[Mesh] OR "radiotherap*"[tiab] OR "radiati*"[tiab] OR "radiosurg*"[tiab] OR "irradiati*"[tiab] OR "x ray therap*" [tiab] OR "radioimmunotherap*"[tiab] OR "immunoradiotherap*"[tiab] OR "intensity modulated therap*"[tiab] OR "Volumetric Modulated Arc Therap*"[tiab] OR "Intensity Modulated Arc Therap*"[tiab] OR "Helical Tomotherap*"[tiab] OR "IMRT"[tiab]	762,050
1	"Head and Neck Neoplasms"[Mesh] OR (cancer[sb] AND ("esophag*"[tiab] OR "face"[tiab] OR "facial*"[tiab] OR "gingiva*"[tiab] OR "head"[tiab] OR "hypopharyn*"[tiab] OR "jaw"[tiab] OR "jaws"[tiab] OR "laryn*"[tiab] OR "lip"[tiab] OR "lips"[tiab] OR "mandib*"[tiab] OR "mouth"[tiab] OR "nasopharyn*"[tiab] OR "neck"[tiab] OR "nose"[tiab] OR "oesophag*"[tiab] OR "oral*"[tiab] OR "oropharyn*"[tiab] OR "otorhinolaryn*"[tiab] OR "palatal*"[tiab] OR "palate"[tiab] OR "palatum"[tiab] OR "paranasal*"[tiab] OR "parathyr*"[tiab] OR "paroti*"[tiab] OR "pharyn*"[tiab] OR "salivar*"[tiab] OR "sublingual*"[tiab] OR "submandib*"[tiab] OR "throat"[tiab] OR "thyroid*"[tiab] OR "tongue"[tiab] OR "tonsil*"[tiab] OR "uadt"[tiab] OR "upper aerodigestive tract"[tiab]))	770,021

Embase 5867, 3744 excluding conference abstracts (15 March 2021)

Search	Embase Query – March 15, 2021	Results
#5	#4 NOT 'conference abstract'/it	3744
#4	#1 AND #2 AND #3	5867
#3	'quality of life questionnaire'/exp OR (('quality of life'/exp OR 'health status'/exp) AND ('health survey'/exp OR 'questionnaire'/exp)) OR ((('quality of life' OR 'qol') NEAR/3 ('assess*' OR 'question*' OR 'survey' OR 'instrument' OR 'index')):ti,ab,kw) OR 'qlq-hn35':ti,ab,kw OR 'qlq-c30':ti,ab,kw OR 'h&n35':ti,ab,kw OR 'uwqolq':ti,ab,kw OR 'neck survey':ti,ab,kw OR 'fact-h&n':ti,ab,kw OR 'oral function*':ti,ab,kw OR 'masticatory function':ti,ab,kw OR 'swallow*':ti,ab,kw OR 'chew*':ti,ab,kw	253572
#2	'radiotherapy'/exp OR 'irradiati*':ti,ab,kw OR 'radiat*':ti,ab,kw OR 'radiotherap*':ti,ab,kw OR 'radiohypophysectom*':ti,ab,kw OR 'radiotreatment*':ti,ab,kw OR 'radioimmunotherap*':ti,ab,kw OR 'immunoradiotherap*':ti,ab,kw OR 'imrt':ti,ab,kw OR 'helical	1105149

	thomotherap*':ti,ab,kw OR ((('bioradiant' OR 'bucky' OR 'radio' OR 'roentgen' OR 'rontgen' OR 'x ray' OR 'volumetric modulated' OR 'intensity modulated') NEAR/3 ('therap*' OR 'radiotherap*' OR 'treatment*')):ti,ab,kw)	
#1	'head and neck tumor'/exp OR 'hnscc':ti,ab,kw OR 'scchn':ti,ab,kw OR (('neoplasm'/exp OR 'adenoma*':ti,ab,kw OR 'anticarcinogen*':ti,ab,kw OR 'blastoma*':ti,ab,kw OR 'cancer*':ti,ab,kw OR 'carcinogen*':ti,ab,kw OR 'carcinom*':ti,ab,kw OR 'carcinosarcoma*':ti,ab,kw OR 'chordoma*':ti,ab,kw OR 'malignan*':ti,ab,kw OR 'melanom*':ti,ab,kw OR 'mesenchymoma*':ti,ab,kw OR 'metasta*':ti,ab,kw OR 'neoplas*':ti,ab,kw OR 'neuroma*':ti,ab,kw OR 'nsclc':ti,ab,kw OR 'oncogen*':ti,ab,kw OR 'oncolog*':ti,ab,kw OR 'paraneoplastic':ti,ab,kw OR 'plasmacytoma*':ti,ab,kw OR 'precancerous':ti,ab,kw OR 'sarcoma*':ti,ab,kw OR 'teratocarcinoma*':ti,ab,kw OR 'teratoma*':ti,ab,kw OR 'tumor*':ti,ab,kw OR 'tumour*':ti,ab,kw) AND ('esophag*':ti,ab,kw OR 'face':ti,ab,kw OR 'facial*':ti,ab,kw OR 'gingiva*':ti,ab,kw OR 'head':ti,ab,kw OR 'hypopharyn*':ti,ab,kw OR 'jaw':ti,ab,kw OR 'jaws':ti,ab,kw OR 'laryn*':ti,ab,kw OR 'lip':ti,ab,kw OR 'lips':ti,ab,kw OR 'mandib*':ti,ab,kw OR 'mouth':ti,ab,kw OR 'nasopharyn*':ti,ab,kw OR 'neck':ti,ab,kw OR 'nose':ti,ab,kw OR 'oesophag*':ti,ab,kw OR 'oral*':ti,ab,kw OR 'oropharyn*':ti,ab,kw OR 'otorhinolaryn*':ti,ab,kw OR 'palatal*':ti,ab,kw OR 'palate':ti,ab,kw OR 'palatum':ti,ab,kw OR 'paranasal*':ti,ab,kw OR 'parathyr*':ti,ab,kw OR 'paroti*':ti,ab,kw OR 'pharyn*':ti,ab,kw OR 'salivar*':ti,ab,kw OR 'sublingual*':ti,ab,kw OR 'submandib*':ti,ab,kw OR 'throat':ti,ab,kw OR 'thyroid*':ti,ab,kw OR 'tongue':ti,ab,kw OR 'tonsil*':ti,ab,kw OR 'uadt':ti,ab,kw OR 'upper aerodigestive tract':ti,ab,kw))	920335

Web of Science 2913 (15 March 2021)

Search	Web of Science Query – March 15, 2021	Results
#4	#1 AND #2 AND #3	2913
	Indexes=SCI-EXPANDED, SSCI, A&HCI, ESCI Timespan=All years	
#3	TS=(((“quality of life” OR “QOL”) NEAR/3 (assess* OR question* OR survey OR instrument OR index)) OR “QLQ-HN35” OR “QLQ-C30” OR “H&N35” OR “UWQOLQ” OR “Neck Survey” OR “FACT-H&N” OR “oral function*” OR “masticatory function” OR “swallow*” OR “chew*”)	110412
	Indexes=SCI-EXPANDED, SSCI, A&HCI, ESCI Timespan=All years	
#2	TS=(“irradiati*” OR “radiat*” OR “radiotherap*” OR “radiohypophysectom*” OR “radiotreatment*” OR “radioimmunotherap*” OR “immunoradiotherap*” OR “IMRT” OR “helical thomotherap*” OR ((“bioradiant” OR “bucky” OR “radio” OR “roentgen” OR “rontgen” OR “x ray” OR “volumetric modulated” OR “intensity modulated”) NEAR/3 (“therap*” OR “radiotherap*” OR “treatment*”)))	1505656
	Indexes=SCI-EXPANDED, SSCI, A&HCI, ESCI Timespan=All years	
#1	TS=(“hnscc” OR “scchn” OR ((“adenoma*” OR “anticarcinogen*” OR “blastoma*” OR “cancer*” OR “carcinogen*” OR “carcinom*” OR “carcinosarcoma*” OR “chordoma*” OR “malignan*” OR “melanom*” OR “mesenchymoma*” OR “metasta*” OR “neoplas*” OR “neuroma*” OR “nsclc” OR “oncogen*” OR “oncolog*” OR “paraneoplastic” OR	507264

	“plasmacytoma*” OR “precancerous” OR “sarcoma*” OR “teratocarcinoma*” OR “teratoma*” OR “tumor*” OR “tumour*”) AND (“esophag*” OR “face” OR “facial*” OR “gingiva*” OR “head” OR “hypopharyn*” OR “jaw” OR “jaws” OR “laryn*” OR “lip” OR “lips” OR “mandib*” OR “mouth” OR “nasopharyn*” OR “neck” OR “nose” OR “oesophag*” OR “oral*” OR “oropharyn*” OR “otorhinolaryn*” OR “palatal*” OR “palate” OR “palatum” OR “paranasal*” OR “parathyr*” OR “paroti*” OR “pharyn*” OR “salivar*” OR “sublingual*” OR “submandib*” OR “throat” OR “thyroid*” OR “tongue” OR “tonsil*” OR “uadt” OR “upper aerodigestive tract”)))	
	Indexes=SCI-EXPANDED, SSCI, A&HCI, ESCI Timespan=All years	

Cochrane 1069 (15 March 2021)

Search	Cochrane Query – March 15, 2021	Results
#1	(hnscc OR scchn OR ((adenoma* OR anticarcinogen* OR blastoma* OR cancer* OR carcinogen* OR carcinom* OR carcinosarcoma* OR chordoma* OR malignan* OR melanom* OR mesenchymoma* OR metasta* OR neoplas* OR neuroma* OR nsclc OR oncogen* OR oncolog* OR paraneoplastic OR plasmacytoma* OR precancerous OR sarcoma* OR teratocarcinoma* OR teratoma* OR tumor* OR tumour*) AND (esophag* OR face OR facial* OR gingiva* OR head OR hypopharyn* OR jaw OR jaws OR laryn* OR lip OR lips OR mandib* OR mouth OR nasopharyn* OR neck OR nose OR oesophag* OR oral* OR oropharyn* OR otorhinolaryn* OR palatal* OR palate OR palatum OR paranasal* OR parathyr* OR paroti* OR pharyn* OR salivar* OR sublingual* OR submandib* OR throat OR thyroid* OR tongue OR tonsil* OR uadt OR upper aerodigestive tract))):ti,ab,kw	50135
#2	(irradiati* OR radiat* OR radiotherap* OR radiohypophysectom* OR radiotreatment* OR radioimmunotherap* OR immunoradiotherap* OR "IMRT" OR ("helical" NEAR/2 thomotherap*) OR (("bioradiant" OR "bucky" OR "radio" OR "roentgen" OR "rontgen" OR "x ray" OR "volumetric modulated" OR "intensity modulated") NEAR/3 (therap* OR radiotherap* OR treatment*))):ti,ab,kw	52055
#3	((("quality of life" OR "QOL") NEAR/3 (assess* OR question* OR "survey" OR "instrument" OR "index")) OR "QLQ HN35" OR "QLQ C30" OR "H&N35" OR "UWQOLQ" OR "Neck Survey" OR "FACT H&N" OR ("oral" NEXT function*) OR ("masticatory" NEXT function*) OR swallow* OR chew*):ti,ab,kw	41293
#4	#1 AND #2 AND #3	1069
